# Molecular Cytogenetic Mapping of Chromosomal Fragments and Immunostaining of Kinetochore Proteins in *Beta*

**DOI:** 10.1155/2009/721091

**Published:** 2009-11-08

**Authors:** Daryna Dechyeva, Thomas Schmidt

**Affiliations:** Institute of Botany, Dresden University of Technology, Zellescher Weg 20 b, 01217 Dresden, Germany

## Abstract

By comparative multicolor FISH, we have physically mapped small chromosome fragments in the sugar beet addition lines PRO1 and PAT2 and analyzed the distribution of repetitive DNA families in species of the section *Procumbentes* of the genus *Beta*. Six repetitive probes were applied, including genotype-specific probes—satellites pTS4.1, pTS5, and pRp34 and a dispersed repeat pAp4, the telomere (TTTAGGG)_n_, and the conserved 18S-5.8S-25S rRNA genes. Pachytene-FISH analysis of the native centromere organization allowed proposing the origin of PRO1 and PAT2 fragments. Comparative analysis of the repetitive DNA distribution and organization in the wild beet and in the addition lines allowed the development of a physical model of the chromosomal fragments. Immunostaining revealed that the PRO1 chromosome fragment binds *α*-tubulin and the serine 10-phosphorylated histone H3 specific for the active centromere. This is the first experimental detection of the kinetochore proteins in *Beta* showing their active involvement in chromosome segregation in mitosis.

## 1. Introduction

The characterization of the genome architecture of higher plants is an important scientific task. One of the most unequivocal approaches to reach this aim is to visualize distinctive chromosomal domains directly by fluorescent-in situ-hybridization (FISH). This method is of supreme efficiency to reveal the physical organization of DNA on plant chromosomes at high resolution. It allows the detection and precise localization of repetitive or single-copy sequences on interphase nuclei, chromosomes in mitosis and meiosis or chromatin fibers. After the first application in wheat [1], FISH was used in plants molecular cytogenetics for the localization of genes, karyotyping and analysis of the physical genome organization [2–4].

A large portion of plant genomes accounts for repetitive DNA [5–8]. Repeats are present in form of sequence duplications up to hundreds of thousands copies [9]. They evolve rapidly in copy number resulting in species-specific variants and/or novel sequence families [10] and are thus crucial for genome evolution [11]. On the other hand, members of many repetitive families show a remarkably high conservation; this ambivalence is a key feature of repeats in genome evolution [12]. The fast evolution leads to a characteristic distribution of the satellites in genomes of closely and distantly related species. While some of these sequences occur in a wide range of plant taxa, others are highly specific. This peculiarity makes repeats a useful tool for comparative studies of plant genomes and for the investigation of evolutionary relationship between plant species [13–16].

Centromeres are essential functional domains of plant chromosomes. They are detectable as primary constrictions or heterochromatic blocks and are responsible for the segregation of the sister chromatids during cell division. The centromere composition was analyzed to different extent in yeast, *Drosophila*, *humans*, *Arabidopsis* [17], rice [18, 19], partially for maize [20] and barley [21]. Plants have regional centromeres, spanning several megabase pairs and are generally composed of species-specific satellite DNA interspersed with retrotransposons, predominantly Ty3-*gyps* [22, 23], but may also contain several genes [24, 25].

The proteins interacting with plant centromere have also attracted attention [26–29]. The nucleosomes of centromeres are characterized by a special H3-like histone CENH3 [30]. The centromere-associated proteins such as CENH3 (mammalian CENP-A), CENP-C and CENP-E in plants, animal and fungi have highly conserved domains [30]. To fulfill its function in cell division, a kinetochore complex is build at the centromeres where the microtubuli of the spindle apparatus are attached [31].

Eukaryotic chromosomes are terminated by specific nucleoprotein complexes—the telomeres. They are important domains responsible for the maintaining of genome stability. Telomeres permit cells to distinguish chromosome ends from double-strand breaks, thus preventing chromosome degradation and fusion [32]. They also participate in the establishment of the synaptonemal complex during meiosis [33]. The first plant telomere was cloned from *Arabidopsis* by Richards and Ausubel [34]. This sequence is highly conserved, consisting of the short repeat motif (TTTAGGG)_n_ arranged in tandem arrays many hundreds of units long [35]. Most dicots have *Arabidopsis*-type telomere, while *Asparagales* possess variant sequences instead [36–38].

*Beta* species provide a suitable system for the comparative study of the nuclear genome composition and evolution. The genus *Beta* contains 14 closely and distantly related species and is subdivided into the sections *Beta*, *Corollinae*, *Nanae* and *Procumbentes* with all cultivars (sugar, fodder and table beet, Swiss chard) exclusively belonging to the section *Beta* [39]. The sugar beet has a genome size of approximately 758 Mbp DNA [39] with at least 63% repetitive sequences [5, 40], and a basic chromosome number of *n* = 9. It is a relatively young crop which origin could be traced back to a few crosses in the 18th century [41]. Therefore, sugar beet has limited genetic variability, and wild beets may provide a valuable pool of genetic resources [42]. To improve the resistance of cultivated beet to biotic and abiotic stress, triploid hybrids were generated by crossing a tetraploid sugar beet with *B*. *procumbens *(2*n* = 18) and *B*. *patellaris *(2*n* = 36) belonging to the section *Procumbentes*. After back-crossing with diploid *B*. *vulgaris*, *nematode*-*resistant* fragment addition lines PRO1 and PAT2 [43, 44] were selected among offspring. Although resistant to pests, the sugar content and biomass production of those hybrids are low. However, these addition lines are a valuable resource for genomic studies.

In this paper, we analyzed the physical organization of the small wild beet chromosome fragments in the two sugar beet mutant lines, PRO1 and PAT2, by multicolor FISH. Pachytene-FISH on meiotic chromosomes was applied to resolve the structure of the wild beet centromeres. Modification of the proteins in the active kinetochore on *B*. *vulgaris* and PRO1 centromeres was demonstrated by immunostaining.

## 2. Materials and Methods

### 2.1. Plant Material

Plants were grown under greenhouse conditions. The following wild beet species were included in this study: *Beta procumbens* (JKI 35336) and *Beta patellaris *(JKI 54753). The seeds were obtained from Federal Research Centre for Cultivated Plants––Julius Kühn Institut, Braunschweig, Völkenrode, Germany, and are now available from Genebank of the Institute of Plant Genetics and Crop Plant Research (IPK), Gatersleben, Germany. The *Beta vulgaris* fragment addition lines PRO1 [43] and PAT2 [44] were obtained from C. Jung (Institute of Crop Science and Plant Breeding, Christian-Albrechts University of Kiel, Germany).

### 2.2. Chromosome Preparation

Mitotic chromosomes were prepared from the meristem of young plants according to Schwarzacher and Heslop-Harrison [45] with some modifications [46] in enzyme solution in citrate buffer containing 6% cellulase *Aspergillus niger*, 0,77% cellulase Onozuka, and 3,0% pectinase *Aspergillus niger.*

Meiotic chromosomes were prepared from anthers by a squashing method [47] with modifications. The buds from the apex of a flower spike having young anthers at early meiosis were used. The 0.45–0.70 mm long anthers were isolated directly from fresh flower buds without fixation or pretreatment and immediately squashed onto a glass slide in 60% acetic acid. Preparations were checked individually for the presence of chromosomes at pachytene and fixed in fresh Carnoy's fixative (methanol : glacial acetic acid = 3 : 1 v/v).

### 2.3. DNA Probes

A set of six repetitive DNA probes representing *Procumbentes*-specific sequences, characterized by different types of organization on plant chromosomes—centromeric, terminal, or dispersed, and an rDNA probe, was used. The satellite probes were clones pTS4.1, pTS5(accession numbers Z50808, Z50809 [48, 49]), and pRp34 (accession number AM076752 [50]), while the clone pAp4 is a dispersed repeat (accession number AJ414552 [51]).

The clone pLt11 consisting of TTTAGGG repeats was used as a telomeric probe [52], and pTa71 from *T. aestivum* was used as an 18S-5.8S-25S rDNA probe (accession number X07841 [53]).

### 2.4. Probe Labeling and FISH

Cloned probes shorter than 3 kb were labeled with biotin-16-dUTP or digoxigenin-11-dUTP by PCR using universal primers. The rDNA probe and the telomeric probe pLt11 were labeled with DIG- or BIO-Nick Translation kits (Roche) following the manufacturer's instructions.

Fluorescent in situ hybridization (FISH) was performed according to Heslop-Harrison et al*.* [54] modified by Schmidt et al. [55]. The microscopy slides were incubated overnight at 37°C and pretreated with 10 ng/l *μ*L of RNase A for 1 hour at 37°C and 50 ng/l *μ*L pepsin for 5 minute at 37°C. Afterwards, the preparations were fixed in freshly prepared 4% formaldehyde solution for 15 minute, dehydrated in ethanol series, and air-dried. However, 30 l *μ*L of the hybridization solution with a stringency of 76% and containing 50% formamide, 20% dextran sulphate, 0.2% SDS, 50 ng/l *μ*L blocking DNA, and 10–100 ng of labelled probes in 2 x SSC were applied. The preparations were covered with plastic cover slips, denatured, and stepwise cooled down in an in situ thermocycler Touchdown (ThermoHybaid) and hybridized overnight at 37°C in a humid chamber. Signals were detected using antibodies coupled to fluorochromes Cy3 (red) or FITC (green). Chromosome preparations were counterstained with DAPI (4′, 6′-diamidino-2-phenylindole) and mounted in antifade solution (CitiFluor).

### 2.5. Immunostaining

Immunostaining was performed according to Houben et al. [56] with modifications for *Beta* species. Root tips were fixed in 4% formaldehyde in microtubule stabilizing buffer (MTSB), washed in MTSB, and treated with enzyme mix consisting of 2.5% pectinase, 2.5% cellulase Onozuka R 10 and 2.5% pectolyase for 30 minute at 37°C. The material was macerated and centrifuged onto a glass slide with a Cytospin 3 (Shandon) at 2000 rpm for 5 minute. The preparations were prefixed in 4% formaldehyde in PBS and blocked with 3% BSA in MTSB/0.2% Tween. Also, 50 l *μ*L of the antibodies solution containing anti-*α*-tubulin (raised in mouse against rabbit, Amersham) and anti-H3 phosphorylated at Ser 10 (polyclonal rabbit, Upstate) were applied to the preparations. After overnight incubation at 37°C, the slides were washed in MTSB three times and the probes were detected with the fluorochrome-conjugated secondary antibodies anti-mouse-FITC (Roche) for anti-*α*-tubulin and anti-rabbit-rhodamin red (Roche) for anti-H3. Unspecific binding of antibodies was removed by washing in MTSB. The preparations were counterstained with DAPI (4′, 6′-diamidino-2-phenylindole) and mounted in antifading solution (CitiFluor).

### 2.6. Image Processing

Examination of slides was carried out with a Zeiss Axioplan2 fluorescence microscope equipped with Filter 01 (DAPI), Filter 02 (Cy3), Filter 09 (FITC), and Filter 25 (DAPI/Cy3/FITC). Photographs were taken on Fujicolor SUPERIA 400 colour print film and negative films were digitized on a Nikon LS-1000 scanner. Alternatively, the images were acquired directly with the Applied Spectral Imaging v. 3.3 coupled with the high-resolution CCD camera ASI BV300-20A.

Immunostaining images were acquired directly with a cooled CCD camera. After three-dimensional deconvolution, the resulting data subsets were merged through the *Z*-axis (DeltaVision).

The images were contrast optimized using only functions affecting the whole image equally and printed using Adobe Photoshop 7.0 software.

## 3. Results

### 3.1. High-Resolution FISH-Mapping of *B. vulgaris* Fragment Addition Lines

To analyze the physical organization of the chromosome fragments in the addition lines PRO1 and PAT2, multicolor FISH with *Procumbentes*-specific and heterologous repetitive sequences was applied. Six repetitive probes (Table 1) were hybridized in situ to chromosomes of PRO1 and PAT2 as well as to the respective donor species of the chromosome fragments *B. procumbens* and *B. patellaris*.

**Figure 1:**
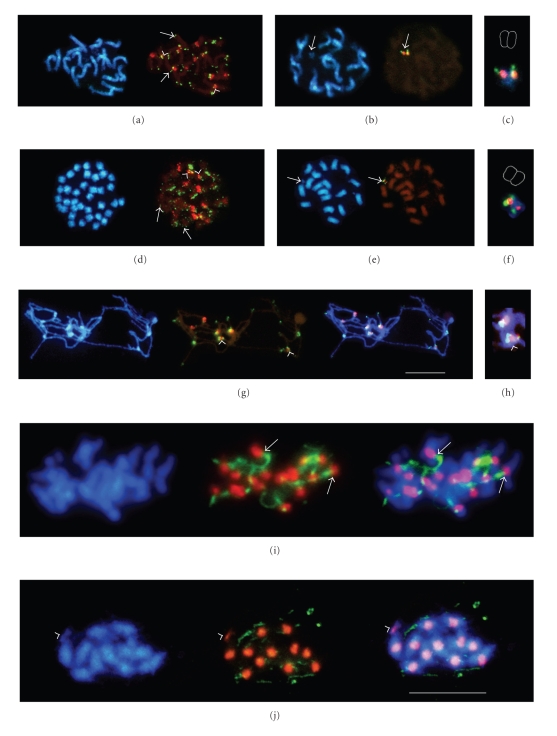
Blue fluorescence in each panel shows the chromosomes stained with DAPI. The scale bar in (G) for the panels A-G and in (J) for the panels I and J represents 10 *µ*m. The chromatids of the chromosome fragments are schematically contoured in panels C and F. FISH with *Procumbentes*-specific satellites pTS4.1 (green) and pTS5 (red) on (A) *B. procumbens*; (B) PRO1; (C) the closed-up overlay of the panel (B); (D) *B. patellaris*; (E) PAT2; (F) the PAT2 fragment. (G) Simultaneous localization of the centromeric probes pTS5 (red) and pTS4.1 (green) on the *B. procumbens* meiotic chromosomes. (H) Close-up from the panel (G). (I-J) Localization of kinetochore proteins in *B. vulgaris* and PRO1 by immunostaining. Microtubuli are visible as green threads. Serine 10-phosphorylated histone H3 produces red signals. The right images are overlays. Microphotographs of the three-dimensional preparation were taken in different focal planes and overlaid. (I) Serine 10-phosphorylated histone H3 labels all centromeres of *B. vulgaris* in mitosis. The sites where the microtubuli of the spindle apparatus are attached to the centromeres are exampled by arrows. (J) PRO1 chromosomal fragment shows a H3S10- phosphorylated signal (arrowhead), thus indicating that its centromere is active in mitosis.

The in situ hybridization of *B. procumbens* with the pericentromeric probe pTS4.1 and the centromeric pTS5 demonstrated that the satellite pTS5 resides on 12 centromeres out of 18, where it is flanked with pTS4.1 (Figure 1(a), exampled by arrowheads). However, pTS4.1 occupies pericentromeric loci of all chromosomes. In contrast to pTS5 strictly confined to the centromeres, it produced weak signals on some intercalary and subterminal sites (Figure 1(a), arrows).

In PRO1, both satellites are detectable only on the chromosomal fragment (Figure 1(b)). pTS5 shows one pair of clear signals on the acrocentric fragment and is bordered by pTS4.1 from one side (Figure 1(c)).

The two centromeric satellites pTS5 and pTS4.1 were hybridized simultaneously to the tetraploid *B. patellaris* (Figure 1(d)). The satellite pTS5 labeled only twelve centromeres out of 36 (Figure 1(d), red). The pTS4.1 hybridization signals of different intensity were detectable in pericentromeric loci of all *B. patellaris* chromosomes (Figure 1(d), green) and on some chromosome ends (arrows).

In PAT2, the *B. patellaris* fragment barely visible after DAPI staining was clearly distinguished after fluorescent in situ hybridization with the genome-specific probes pTS5 and pTS4.1 (Figure 1(e), arrows). Figure 1(f) shows that the centromeric pTS5 (red) is flanked by pTS4.1 (green) producing two pairs of signals. This is strikingly in contrast with the hybridization pattern on the PRO1 chromosomal fragment, where the stretch of pTS4.1 is detectable only on one side of the centromeric pTS5 array (Figure 1(c)).

To achieve a higher resolution of the physical organization of the two centromeric satellites, a double-target in situ hybridization with pTS4.1 and pTS5 was performed on meiotic chromosomes of *B. procumbens* (Figure 1(g)). The chromosomes at pachytene are far less condensed than at mitosis, but still preserve their morphology. The chromatin at this stage of the cell cycle enables a higher resolution and is especially suitable for the simultaneous detection of adjacent sequences. The experiment on meiotic spreads showed that on some centromeres the satellite pTS5 is flanked by the pTS4.1 (Figures 1(g) and 1(h), arrowheads), while on the others the pTS4.1 borders pTS5 only from one side.

**Figure 2:**
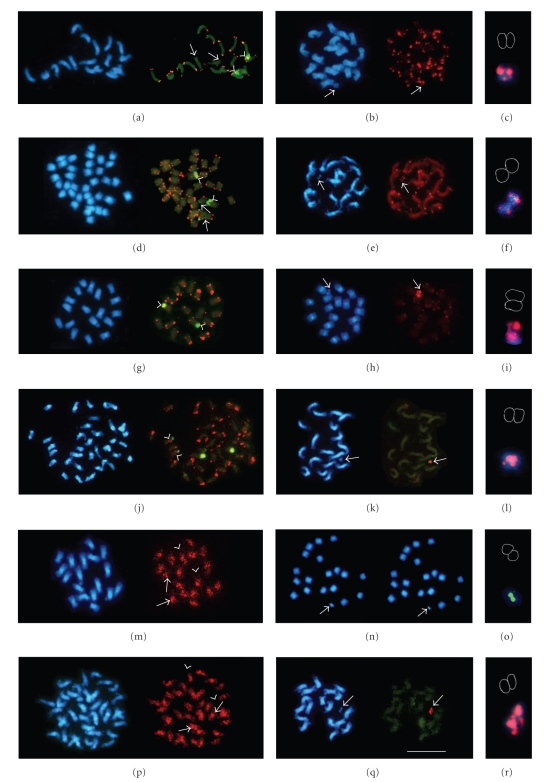
Blue fluorescence in each panel shows the chromosomes stained with DAPI. The scale bar for left and central panels in (Q) represents 10 *µ*m. Green fluorescence shows hybridization of the ribosomal gene probe pTa71. The chromatids of the chromosome fragments are schematically contoured in right panels. FISH with the telomeric probe (TTTAGGG)n (red) and on (A) *B. procumbens*; (B) PRO1;. (C) PRO1 chromosome fragment; (D) *B. patellaris*; (E) PAT2; (F) PAT2 chromosome fragment. The subtelomeric satellite repeat pRp34 (red) cloned from *B. procumbens* is found on (G) *B. procumbens*; (H) PRO1 and its chromosome fragment (I); (J) *B. patellaris*; (K) PAT2; (L) close-up of the panel (H). The dispersed repeat pAp4 specific to the *Procumbentes* localized by FISH on (M) *B. procumbens*; (N) on the PRO1 chromosome fragment; (O) close-up of the panel (N); (P) *B. patellaris*; (Q) on PAT2 is limited to the chromosome fragment, where it shows a weak dispersed distribution (R).

The hybridization of a *B. procumbens* prometaphase spread with the telomeric probe pLT11 produced clear double signals with variable intensity on all chromosome ends (Figure 2(a), red) with the exception of a pair of chromosomes where signals were detected on one arm only (Figure 2(a), arrows). The 25S-18S ribosomal gene fragment pTa71 was used as a second probe (Figure 2(a), green). The rDNA in *B. procumbens* forms a clearly visible distal secondary constriction [57]. In this species, the rDNA array is adjacent to the telomere (Figure 2(a), arrowheads) as has been shown previously (Dechyeva and Schmidt [50]).

On the PRO1 metaphase spread, the telomeric DNA was detectable on all sugar beet chromosomes as well as on both ends of the chromosome fragment (Figure 2(b), red) where it produced a strong pair of signals on one end and a very weak one on the opposite arm (Figure 2(c), red).

The telomeric probe pLT11 labeled the ends of all *B. patellaris* chromosomes relatively uniformly (Figure 2(d), red). The simultaneous hybridization with the ribosomal gene fragment pTa71 produced two strong signals, two weak and two barely visible additional signals, all at the subterminal positions (Figure 2(d), green). The telomeres found adjacent to the larger rDNA array (Figure 2(d), arrowheads). The only chromosome, arms where pLT11 was not detectable, were those with minor pTa71 site (Figure 2(d), arrows).

As expected, all PAT2 chromosomes demonstrated telomeric signals (Figure 2(e), red). The signals on the wild beet added fragment were nearly as intense as those on sugar beet chromosomes (Figure 2(e), arrows). The chromosomal fragment has two pairs of clear signals, evidently on its both ends (Figure 2(f), red).

The localization of the telomeric sequences on the chromosome fragments was complemented by FISH with the subtelomeric satellite pRp34. The probe labeled all but two chromosomal ends of the wild beet *B. procumbens* (Figure 2(g), red), including those harboring rRNA genes (Figure 2(g), green, arrowheads). In PRO1, the sugar beet chromosomes were labeled with this *B. procumbens*-derived satellite much weaker than the wild beet fragment (Figure 2(h), red, arrow). The two pairs of the pRp34 signals on the fragment have different strengths (Figure 2(i), red).

The subtelomeric probe pRp34 was detected on one or both arms of all except two *B. patellaris* chromosomes in subtelomeric positions producing signals of various intensities (Figure 2(j), red). It is noteworthy that pRp34 signals were also found adjacent to the minor sites of the ribosomal genes (Figure 2(j), green, arrowheads). Both ends of the PAT2 chromosomal fragment showed the subtelomeric satellite signals, one weaker than the other (Figure 2(k), arrow, and Figure 2(l)).

The *Procumbentes*-specific repeat pAp4 was dispersed over all *B. procumbens* chromosomes (Figure 2(m), red). The repeat is amplified in intercalary and pericentromeric heterochromatic regions (Figure 2(m), arrows), but mostly excluded from distal euchromatic regions (Figure 2(m), arrowheads). In PRO1, the dispersed repeat pAp4 is not detectable on sugar beet chromosomes and is only found on *B. procumbens* fragment (Figure 2(n), green, arrows), where it labels both chromatids (Figure 2(o)).

The dispersed repeat pAp4 was scattered over all *B. patellaris* chromosomes (Figure 2(p), red). Examples of the reduction of the repeat in some centromeres are indicated with arrows, while examples of the exclusion from euchromatin are shown by arrowheads (Figure 2(p)). On PAT2, the repeat produced 3-4 pairs of relatively weak signals exclusively along the chromosome fragment (Figure 2(q), arrow, and Figure 2(r)) forming a dispersed pattern similar to that on intact *B. patellaris* chromosomes.

### 3.2. Detection of Phosphorylation in Histone H3 at Centromeres of *B. vulgaris* and PRO1 by Immunostaining

To get an insight into the structure and function of the kinetochore, the proteins characteristic for active centromeres were observed on metaphase preparations of *B. vulgaris* and the fragment addition line PRO1.

Mitotic beet cells were immunostained with polyclonal antibody against serine 10-phosphorylated histone H3 raised in rabbit [56]. This N-terminal modification of histone H3 is only found in functional pericentromeric chromatin [58]. The antibody against *α*-tubulin raised in mouse against rabbit allows visualizing the microtubuli [59] which attach as important functional parts to the active kinetochore.

Proteins in immunostaining probes should preserve their structure resulting in a three-dimensional shape of the nuclei. Such preparations cannot be successfully analyzed by conventional epifluorescence microscopy. Consequently, computational deconvolution microscopy was applied. The immunostaining with the antibody against histone H3 phosphorylated at serine 10 demonstrated that the centromeric histones of *B. vulgaris* (Figure 1(i)) and PRO1 (Figure 1(j)) in metaphase are modified by phosphorylation at the N-terminal serine 10. The sites appeared as bright red signals localized at the DAPI-positive centromeric regions. The microtubuli were detectable as green threads. It was clearly visible at some loci that the microtubuli are attached to the centromeric sites (Figure 1(i), arrows). Remarkably, the PRO1 acrocentric chromosomal fragment also showed a clear H3 S10-phosphorylated signal (Figure 1(j), arrowhead).

## 4. Discussion

Sugar beet is an important agricultural crop, and the results of genome research in this species might be important to the practical implementation in green biotechnology. Currently, a fine-resolution physical map is under construction and a genome-sequencing project is carried out in the framework GABI–Genome Analysis in Biological System Plant (http://www.gabi.de/) aiming to unravel the genome composition of this crop species. Interspecific hybrids and addition lines of *B. vulgaris* are a valuable starting material for plant breeders and an interesting object for fundamental studies on plant genome composition and evolution [60–62]. The application of genome-specific repetitive probes isolated from the wild beet *B. procumbens* in combination with repetitive DNA sequences conserved among plant species enabled to map the chromosome fragments of the *B. vulgaris* addition lines PRO1 and PAT2.

### 4.1. Molecular Cytogenetics of the Wild Beet Species

Hybridization of *B. patellaris* with pTS5 (Figure 1(d), red) suggests that this species might be an allopolyploid: the pTS5 gave 12 signals of different intensity similar to the pattern in *B. procumbens*. It is tempting to assume that one haploid set of chromosomes of the *B. patellaris* genome is indeed derived from *B. procumbens*, while the remaining 18 chromosomes originate from another, yet unidentified species. When probed with the subtelomeric pRp34, *B. patellaris* (Figure 1(j), red) in contrast to *B. procumbens* (Figure 1(g), red) did not produce visible signals on the chromosome ends carrying the rDNA genes (Figure 1(j), green). On the contrary, the subtelomeric pRp34 signals were detectable proximal to weaker pTa71 signals (Figure 1(g), arrowheads), most likely caused by an inversion of the rRNA gene array. This is another indication that *B. procumbens* is not the only species that participated in *B. patellaris* polyploidization. Similarly, hybridization of the allopolyploid *Nicotiana rustica* with the satellite NUNSSP specific to the parental U-genome (*N. undulata*) allowed distinguishing chromosomes originating from the different tobacco species, *N. paniculata* (P-genome) [63]. Recent studies on allotetraploid *Gossypium hirsutum* in comparison to the model diploid progenitors, *G. arboreum* and *G. raimondii*, revealed possible mechanisms of “genomic downsizing” in polyploids [64].

Ribosomal DNA genes in eukaryotes are tandemly arranged in thousands of copies. They reside at the chromosomal loci known as nucleolus organizer regions (NORs) [65]. These genes are highly conserved in plants and other eukaryotes. Therefore, it was expected that the heterologous 18S-5.8S-25S rDNA probe pTa71 isolated from wheat [53] produced strong hybridization signals at the secondary constriction of *B. procumbens* (Figure 1(i), arrowheads). The domains harboring rRNA genes are recognizable as prominent DAPI-positive structures located distally on two chromosomes [49, 57]. For polyploids, it has been reported that only one set of parental rRNA genes is preferentially functional: expression of rDNA of rye origin is suppressed in amphiploid triticale, and only the 1B- and 6B-rDNA from wheat is functional [66]. Similar rRNA gene silencing was observed in the natural allotetraploid *Arabidopsis suecica* and the synthetic hybrid of its progenitors *A. thaliana* and *A. arenosa* (*Cardaminopsis arenosa*) [67]. This epigenetic phenomenon, observed in many animals, like *Drosophila* and *Xenopus* [68], and plants, like *Crepis* [69], *Aegilops* x *Triticum* hybrids [70], *Brassica* [71], is known as nucleolar dominance [72]. Not only most natural polyploids possess one predominant 18S-5.8S-25S nuclear ribosomal DNA homolog in their genome; the studies on artificial interspecific hybrids suggested that in some plants, like *Glycine*, most or all repeats at one homeologous locus have been lost [73]. It can be speculated that in the tetraploid species *B. patellaris*, the two strong hybridization signals most likely correspond to functional rDNA loci (Figure 2(d), arrowheads) which can be shown by silver staining. The weak green crosshybridization signals (Figure 2(d), arrows and Figure 2(j), arrowheads) may correspond to the rDNA loci from the repressed copies of the chromosome set, which is still recognizable by the heterologous probe used in this experiment. No pTa71 signals were detectable either on PRO1 or on PAT2 chromosomal fragments (not shown).

### 4.2. Development of a Physical Model of the PRO1 and PAT2 Chromosomal Fragments

The addition line PRO1 [43] has a 6–9 Mbp large fragment of the *B. procumbens* chromosome [61]. The chromosome mutant PAT2 has a smaller fragment originating from *B. patellaris* [44]. Both chromosome fragments are stably transmitted in mitosis and hence should have a functional centromere. FISH-mapping of such small chromosomes is challenging, for comparison, the chromosomes of *A. thaliana* have a size of approximately 25 Mbp (TAIR, http://www.arabidopsis.org/) and are regarded as a difficult subject for conventional FISH.

Previous analysis of the long-range organization of centromeres in the wild beet *B. procumbens* allowed the development of a structural model of a plant centromere [74, 75]. According to this model, the centromeric satellite pTS5 forms a large array which is flanked by the arrays of a nonhomologous pericentromeric satellite pTS4.1. These arrays, representing the majority of centromeric and pericentromeric DNA, are interspersed with Ty3-*gypsy*-like retrotransposons *Beetle*1 and *Beetle*2 and remnants or rearranged copies thereof as shown by BAC analysis and FISH on *B. procumbens* chromosomes [23, 61].

To reveal the possible origin of the PRO1 and PAT2 chromosomal fragments, the organization of the centromere-specific satellites pTS4.1 and pTS5 was studied in detail. Hybridization of pTS5 and pTS4.1 on chromosomes of *Procumbentes* species resulted in a unique pattern on each centromere, thus allowing to classify the centromeres in those having (a) only pTS4.1, (b) both satellites present with signals of equal intensity, and (c) where pTS5 was much stronger than pTS4.1 (Figures 1(a) and 1(d)).

In PRO1, pTS5 labels one end of the acrocentric fragment, bordered by adjacent pTS4.1 array from one side only (Figure 1(c)). Gindullis et al. [75] suggested that the PRO1 fragment may be a result of a chromosome breakage within the centromeric pTS5 block which is flanked with pTS4.1 from both sides (examples of this type of centromere are indicated with arrowheads in Figures 1(a), 1(g), and 1(h)). Alternatively, the PRO1 fragment could originate from one of the chromosomes where the centromeric satellite pTS5 region is bordered by pericentromeric pTS4.1 only from one side (Figure 1(h)). In PAT2, the pTS5 block on the wild beet chromosomal fragment is flanked with pTS4.1 arrays from both ends (Figure 1(f)), which implies that two breaks have occurred within the pericentromeric region. Hence, the most likely donators of the PAT2 chromosome fragment are one of the chromosomes exampled on Figure 1(d) by arrowheads.

The telomeres protect the chromosome ends from degradation. Both fragment addition lines PRO1 and PAT2 arose spontaneously in the offspring of *B. vulgaris * x * B. procumbens* or *B. vulgaris * x * B. patellaris* triploid hybrids back-crossed with diploid *B. vulgaris* [76]. Such chromosome fragments resulting from chromosomal breakage usually have instable ends which tend to fuse. Alternatively, breakpoints may be stabilized by the phenomenon known as “healing of broken ends,” first described and discussed by McClintock [77]. However, healing of broken ends by de novo telomere formation is mostly restricted to meristematic tissue or undifferentiated cells, but is low or undetectable in mature differentiated tissues [78]. Moreover, such newly synthesized telomeres contain a considerable amount of atypical sequence units, as was shown for wheat [79].

Thus, it was important to find out whether PRO1 and PAT2 chromosome fragments indeed possess telomeres ensuring their stability. Therefore, the next probes tested on the sugar beet hybrids PRO1 and PAT2 were those located at the chromosome ends: the *Arabidopsis* telomeric probe pLT11, the subtelomeric satellite pRp34-179 originating from *B. procumbens*, and the 25S-18S rDNA probe pTa71 from wheat. The telomere and the pRp34 were clearly visible as pairs of fluorescent signals on both ends of the PRO1 (Figures 2(c) and 2(i)) and PAT2 (Figures 2(f) and 2(l)) fragments. The subtelomeric satellite pRp34 was found on both ends of the fragments PRO1 (Figure 2(i)) and PAT2 (Figure 2(l)). The two signals on the PRO1 and PAT2 chromosomal fragments indicate that not only the telomeres of the added fragments seem to be intact but also other components of the terminal chromosomal regions are present. Since not only the *Arabidopsis*-type telomeric sequence but also the subtelomeric satellites are preserved, it is possible to assume that the fragments evolved by deletions resulting in the loss of considerable amount of intercalary chromatin.

Additional signals of the subtelomeric satellite pRp34-179 on sugar beet chromosomes (Figures 2(e) and 2(h)) are due to cross-hybridization with the homologous subtelomeric satellite pAv34 from *B. vulgaris* belonging to the same repetitive DNA family as pRp34 [50]. However, these signals are relatively weak because of the relatively low sequence similarity between pAv34 and pRp34 (58.9%). The stronger signals on *B. vulgaris* chromosomes may correspond to the pAv34 sequence subsets more similar to pRp34, thus indicating chromosome-specific amplification of the subtelomeric satellite variations. The reason was most likely the divergence between the pRp34 from *B. procumbens* and pAv34 from *B. vulgaris* which share 56.8–60.7% similarity [50]. In this in situ experiment, hybridization stringency was 76%, which was too high to detect all the copies of pAv34 on *B. vulgaris *chromosomes with pRp34-179 as probe. Emergence of chromosome-specific DNA is known for human alpha satellite, where ancestral sequences have evolved into a number of chromosome-specific families, presumably by cycles of interchromosomal transfers and subsequent amplification leading to intrachromosomal sequence homogenization [80]. Similar divergence of satellite subfamilies into chromosome-specific subsets have been observed in plants. Subtelomeric repeats with chromosome-specific distribution may play a role in the recognition of homologous chromosome ends and have been suggested to be part of a complex chromosome end structure [52]. The analysis of telomeres and adjacent sequences on rye chromosomes showed that they are able to evolve in copy number rapidly [81]. Despite the fact that maize centromeres are all composed of the same related elements, the differences in composition and mutual arrangements of those elements provide each centromere with a unique molecular structure [82]. Similarly, representatives of the *Sau*3AI satellite family I of *B. procumbens* also form the subfamily pTS6 which has a different chromosomal position [49].

The presence of the dispersed repetitive family pAp4 specific for the *Procumbentes* genomes in PRO1 and PAT2 chromosome fragments in well-detectable numbers allows to conclude that the chromosomal fragments also posses intercalary chromatin, which must be essential as a lateral support for the centromeric activity and hence ensure stability of the chromosomal fragments [83].

**Figure 3:**
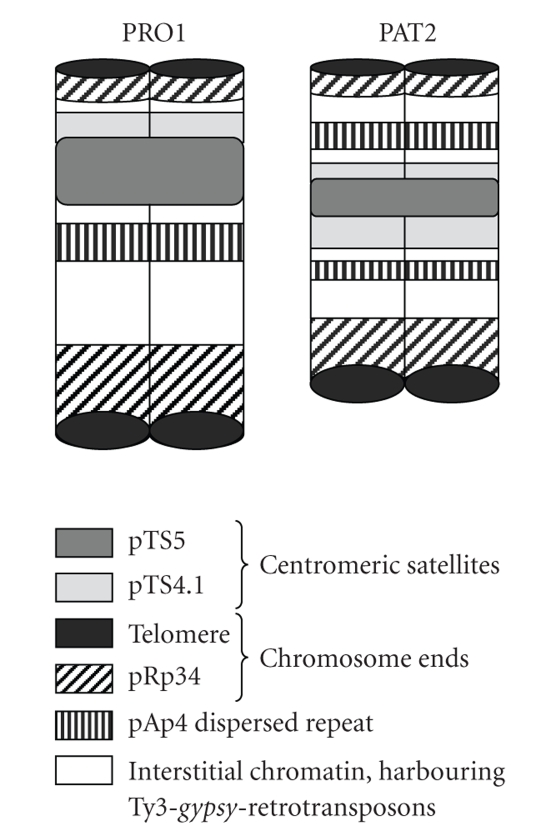
Structural model of the PRO1 and PAT2 chromosomal fragments. Both chromosome fragments are represented according to the distribution patterns of the repetitive DNA sequences mapped by FISH.

The allocation of repetitive probes on PRO1 and PAT2 by FISH enabled to propose origins and to develop physical models of the chromosome fragments (Figure 3). The wild beet fragments in PRO1 and PAT2 contain most major types of repetitive DNA characteristic for intact chromosomes of the wild beet species *B. procumbens* and *B. patellaris*. They include arrays of two centromeric and pericentromeric satellites dispersed repetitive sequences and are terminated by subtelomeric satellite and the telomere protecting their ends from degradation.

The experiments performed in this study demonstrated that the PRO1 fragment is acrocentric. The size of the centromeric pTS5 satellite array has been estimated by fiber FISH to be 115 kb [61]. Wild type *B. procumbens* chromosomes are metacentric, submetacentric, or acrocentric (Figure 1(a)), their centromeric satellite arrays spanning 157–755 kb [74]). In metacentric or submetacentric PAT2 fragment, the centromeric array is even smaller––only about 50 kb estimated by pulsed-field gel electrophoresis [61]. The telomere and the subtelomeric satellite pRp34 were also detected on the ends of the chromosomal fragments although amplified to a different extent. However, the transmission rate of the monosomic PRO1 and PAT2 fragments in meiosis is lower than expected 50%, reaching 34.8% maximal for PRO1 [44]. The fact that the chromosome fragment of PAT2 contains a centromeric pTS5 satellite block flanked by pericentromeric satellite pTS4 arrays as well as the telomere and the subtelomeric satellite repeat (Figure 3) leads to the conclusion that there might be other factors effecting stable transmission of this wild beet fragment in meiosis. It was shown that barley chromosomal fragments can be normally transmitted through meiosis in wheat genetic background even without typical centromeric repeats [84]. On the other hand, the field bean minichromosome DI-VI containing a wild-type centromere and comprising approximately 5% of the haploid metaphase complement suffered loss during meiosis (66% loss in hemizygous condition, which is similar to PRO1 and PAT2), while the minichromosomes comprising approximately 6% of the genome were already stably segregating [83]. However, PRO1 and PAT2 chromosomal fragments are estimated to comprise only 0.8–1.2% of the 758 Mb of sugar beet haploid genome. Field bean minichromosomes of similar proportion of 1% of the haploid genome size were stably transmitted through mitosis, but not meiosis. It is supposed that lack of additional genomic DNA serving as lateral support of centromeres or insufficient bivalent stability due to the incapability of chiasma formation could be the reasons of lower transmission of the very small chromosome fragments, even though they possess the centromere and the telomeres [83]. The data generated by these experiments demonstrate that FISH is a unique method in genome analysis including comparative studies giving an insight into details of the physical organization of DNA sequences on chromosomes as small as 6–9 Mbp.

### 4.3. Kinetochore Proteins in the *B. vulgaris* Hybrid PRO1

There is no conserved DNA sequence responsible for the centromeric function in higher plants [11, 17, 21, 75, 84]. Recently, it has been shown that barley isochromosomes lacking typical centromeric sequences were normally transmitted through mitosis and meiosis [83]. However, the establishment of a centromere must involve the deposition of the centromeric histone H3 variant designated CENH3. This protein is generally viewed as the core of the centromeric complex linking centromeric DNA with the proteins of inner kinetochore. In maize, CENH3 binding to centromeric retrotransposons (CRMs) and satellite repeats (CentC) was shown by immunoprecipitation [85]. A study of rice chromosome 8 centromere (*Cen*8) indicated also that the transcribed genes are interspersed with CENH3 binding sites [25]. Although kinetochores differ in morphology from species to species, recent data have established that an important group of kinetochore proteins is conserved from *Saccharomyces cerevisiae* to humans [58, 86]. It was shown by indirect immunofluorescence that the kinetochore elements of higher plants are conserved even between very distantly related taxa like monocots and dicots [28]. Antibodies against the partial human proteins CENP-C, CENP-E, and CENP-F and against maize CENP-C recognized the centromeric regions of mitotic chromosomes of *Vicia faba* and *Hordeum vulgare* [87].

An important step during the formation of a functional kinetochore is the phosphorylation of the pericentromeric histone H3 [29]. This posttranslational chromatin modification is evolutionarily conserved in plants and animals [58]. The changes in the level of phosphorylation of serine 10 in CENH3 correspond to changes in the cohesion of sister chromatids in meiosis in maize [88]. In *Secale cereale*, *Hordeum vulgare*, and *Vicia faba*, the phosphorylation of the pericentromeric histone H3 at serine 10 correlates with the chromosomes condensation in mitosis [56].

The fluorescent immunostaining of centromere-associated proteins in the fragment addition line PRO1 allowed the comparison of the histone H3 phosphorylation patterns of *B. vulgaris* chromosomes and the *B. procumbens* chromosome fragment in mitosis, elucidating the behavior of the centromeres originating from different species in a single dividing cell. The results demonstrated that the heterologous antibody against serine 10-phosphorylated histone H3 recognized sugar beet kinetochores (Figures 1(i) and 1(j), red). The visualization of the microtubuli with anti-*α*-tubulin (Figures 1(i) and 1(j), green) demonstrated that the chromosomes are attached to the spindle apparatus during mitosis (Figure 1(i), arrows). For oat-maize chromosome addition lines it was shown that the introgressed maize chromosomes do not express their own CENH3 but rather utilize CENH3 available from the host genome [89]. Although species-specific CENH3-antibodies are not yet available for *Beta*, the heterologous antibody against Ser10-phosphorylated centromeric histone H3 readily immunolabelled the centromere of the PRO1 chromosome fragment at mitosis (Figure 1(j), arrowhead). Since mitotic transmission of the PRO1 wild beet fragment is normal, and meiotic transmission is only slightly reduced [76], it is reasonable to assume that the kinetochore proteins expressed by *B. vulgaris* must recognize also the centromere of the added fragment and that the fragment may utilize CENH3 similarly to maize chromosomes in oat background.

The immunostaining experiment with the antibodies against serine 10-phosphorylated histone H3 and *α*-tubulin gave the first insight into the centromeric function in the *B. vulgaris* fragment addition line PRO1. Further studies on this unique material combining the functional centromeres with the different molecular composition from two distantly related species would shed light on the conservation of centromeric components in higher plants.

## Figures and Tables

**Table 1:** Repetitive probes used for the characterization of the fragment addition lines PRO1 and PAT2.

Probe	Origin	Length, bp	Sequence type	Accession	Reference
Satellite					
pTS4.1	*B. procumbens*	312	*Sau*3AI restriction satellite	Z50808	Schmidt et al. 1990 [48]
pTS5	*B. procumbens*	153–160	*Sau*3AI restriction satellite	Z50809	Schmidt and Heslop-Harrison 1996 [49]
pRp34	*B. procumbens*	352–358	*Rsa*I restriction satellite	AM076755	Dechyeva and Schmidt 2006 [50]

Dispersed					
pAp4	*B. procumbens*	1353-1354	*Alu*I repeat	AJ414552	Dechyeva et al. 2003 [51]

Telomere					
pLT11	*A. thaliana*	not tested	telomeric repeat	not entered	Vershinin et al. 1995 [52]

Ribosomal genes					
pTa71	*T. aestivum*	4642	25S-18S gene fragment with spacer	X07841	Barker et al. 1988 [53]
